# Glycerine as an Excipient

**Published:** 1885-03

**Authors:** 


### Glycerine as an Excipient.

The employment of glycerine in pharmacy is becoming more and more extensive every day, owing in great measure to its property of dissolving various medicinal substances. It is thus very generally used instead of lard or oil in a number of cosmetic preparations. It makes an elegant excipient, but, unfortunately, glycerine will not penetrate the pores of the skin, and prevents also the absorption of any drugs which it may hold in solution. Dr. P. Vigier has recently made some experiments upon himself, which are very simple and may be repeated and verified by anyone, which show in a striking manner the uselessness of employing glycerine as an excipient when absorption of the active ingredient is desired (*Memorabilien,* November 20, 1884). He was never able to detect iodine in the urine after repeated inunctions of a solution of potassium iodine in glycerine (one part to three), but, when the glycerine was replaced by a fatty substance, the presence of iodine in the urine was at once manifest. A five per cent. solution of muriate of morphia in glycerine rubbed into the skin produced not the slightest narcotic effect. A one per cent. solution of sulphate of atrophine applied for six hours to the temples caused no dilatation of the pupils. Still more striking was an experiment made with corrosive sublimate. A five per cent. solution of this substance in glycerine was rubbed strongly upon the skin, yet produced not even a reddening of the surface, nor was mercury to be detected in the urine. The result of this experiment leads Dr. Vigier to recommend the employment of a glycerine solution instead of the mercurial ointment as a parasiticide, since with the use of the latter there is always more or less absorption of the drug.—*British Medical Journal.*

